# PLGA and PDMS-based *in situ* forming implants loaded with rosuvastatin and copper-selenium nanoparticles: a promising dual-effect formulation with augmented antimicrobial and cytotoxic activity in breast cancer cells

**DOI:** 10.3389/fphar.2024.1397639

**Published:** 2024-06-04

**Authors:** Amr Maged, Mostafa Mabrouk, Hanzada T. Nour El-Din, Lamyaa Osama, Shaimaa M. Badr-Eldin, Azza A. Mahmoud

**Affiliations:** ^1^ Pharmaceutics and Pharmaceutical Technology Department, Faculty of Pharmacy, Future University in Egypt, New Cairo, Egypt; ^2^ Pharmaceutical Factory, Faculty of Pharmacy, Future University in Egypt, New Cairo, Egypt; ^3^ Refractories, Ceramics and Building Materials Department, National Research Centre, Giza, Egypt; ^4^ Microbiology and Immunology Department, Faculty of Pharmacy, Cairo University, Cairo, Egypt; ^5^ Pharmaceutics Department, Faculty of Pharmacy, King Abdulaziz University, Jeddah, Saudi Arabia; ^6^ Center of Excellence for Drug Research and Pharmaceutical Industries, King Abdulaziz University, Jeddah, Saudi Arabia

**Keywords:** breast cancer, in-situ forming implants, rosuvastatin calcium, copper-selenium nanoparticles, PLGA, antimicrobial activity, healthcare

## Abstract

Breast cancer is among the most prevalent tumors worldwide. In this study, *in-situ* forming implants (ISFIs) containing rosuvastatin calcium were prepared using three types of poly (D, L-lactic-co-glycolic acid) (PLGA), namely, PLGA 50/50 with ester terminal and PLGA 75/25 with ester or acid terminal. Additionally, polydimethylsiloxane (PDMS) was added in concentrations of 0, 10, 20, and 30% w/v to accelerate matrix formation. The prepared ISFIs were characterized for their rheological behaviors, rate of matrix formation, and *in-vitro* drug release. All the prepared formulations revealed a Newtonian flow with a matrix formation rate between 0.017 and 0.059 mm/min. Generally, increasing the concentration of PDMS increased the matrix formation rate. The prepared implants’ release efficiency values ranged between 46.39 and 89.75%. The ISFI containing PLGA 50/50 with 30% PDMS was selected for further testing, as it has the highest matrix formation rate and a promising release efficiency value. Copper-selenium nanoparticles were prepared with two different particle sizes (560 and 383 nm for CS1 and CS2, respectively) and loaded into the selected formulation to enhance its anticancer activity. The unloaded and loaded implants with rosuvastatin and copper-selenium nanoparticles were evaluated for their antibacterial activity, against Gram-positive and negative microorganisms, and anticancer efficacy, against MCF-7 and MDA-MB-231 cell lines. The results confirmed the potency of rosuvastatin calcium against cancer cells and the synergistic effect when loaded with smaller particle sizes of copper-selenium nanoparticles. This formulation holds a considerable potential for efficient breast cancer therapy.

## Graphical Abstract

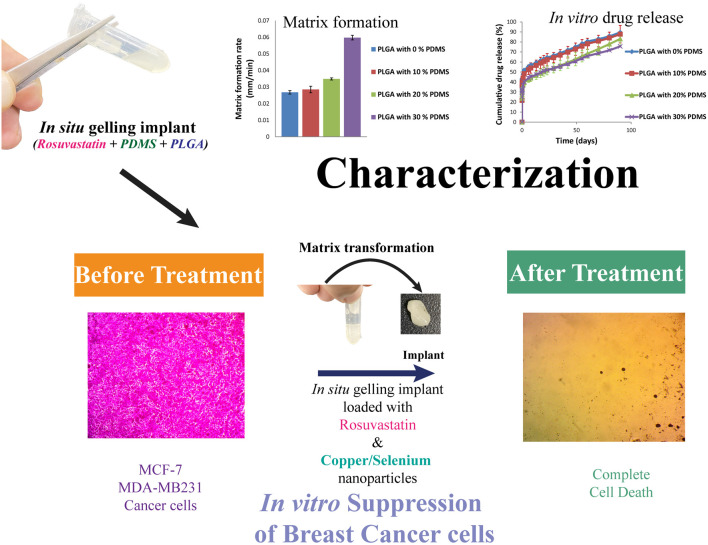

## 1 Introduction

Cancer poses a significant threat to life. Breast cancer is currently the most common type globally, accounting for approximately 12.5% of new reported cases each year (Breast Cancer, 2023). Surgical excision, accompanied by adjuvant chemotherapy or radiation therapy, remains the primary treatment for solid tumors (Maluccio and Covey, 2012; Dieli-Conwright et al., 2014; Muhammed Ashraf et al., 2021).

Statins are a common class of medications used to reduce the cholesterol levels. They primarily work by blocking the rate-limiting stage in cholesterol synthesis (Hess et al., 2018). While effectively lowering blood cholesterol levels, their mechanistic effect extends to cell signaling, potentially impacting cancer cell proliferation (Beckwitt et al., 2018). This link between cholesterol metabolism and cancer cell signaling has led to investigations into the potential anticancer effects of statins. Studies have demonstrated the effect of statins on lowering breast cancer recurrence rates (Ahern et al., 2011; Manthravadi et al., 2016; Sakellakis et al., 2016; Sim et al., 2022). Moreover, the utilization of statins has been shown to significantly reduce Breast Cancer-Specific Mortality (BCSM) (Murtola et al., 2014; Cardwell et al., 2015; Mc Menamin et al., 2016; Liu et al., 2017; Nowakowska et al., 2021).

Rosuvastatin calcium, a renowned statin, has about 20% absolute bioavailability and it effectively lowers lipid levels in the body (CRESTOR, 2005; Maged et al., 2020). By inhibiting some of the enzymes required for cell growth, rosuvastatin holds potential in limiting the growth of tumor cells. Therefore, the administration of rosuvastatin after the surgical removal of cancerous tissue may aid in eliminating any residual tumor (Kamel and Mahmoud, 2013). This can be achieved by localized drug delivery of rosuvastatin to the tumor site achieving high initial drug levels that can be further regulated based on the utilized drug delivery system.

Rosuvastatin was reported to inhibit and suppress cancer growth in androgen dependent prostate cancer (Deezagi and Safari, 2020). Additionally, the use of rosuvastatin-loaded polylactic-co-glycolic acid (PLGA) nanoparticles showed promising effect in treatment of hepatic cancer (Aldalaen et al., 2019). Furthermore, by inhibiting the arginase enzyme activity and reducing the ornithine levels, which serve as precursors to polyamines, rosuvastatin might offer preventive benefits against the onset of breast cancer (Erbas et al., 2015). On another front, statins were reported to have a variety of effects, other than their previously mentioned antihyperlipidemic and anticancer activity, including immunomodulatory, antioxidative, anticoagulant properties and antibacterial potency against both Gram-positive and Gram-negative organisms (Welsh et al., 2009; Masadeh et al., 2012).

One of the extensively studied elements in cancer prevention and immune enhancement is selenium (Se). It is a micronutrient known for its exceptional pharmacological and physiological properties as well as important biological functions (Garbo et al., 2022). Researchers have extensively reviewed the chemopreventive and anticancer activities of Se and Se-containing compounds, as well as its implications in human health and nutrition (Navarro-Alarcon and Cabrera-Vique, 2008; Ali et al., 2018; Avery and Hoffmann, 2018; Valente et al., 2021). Various forms of selenium compounds exhibit biological activity, including nanoparticles, selenium salts and selenoproteins (Vinceti et al., 2017; Liu et al., 2018; Khurana et al., 2019). Selenium nanoparticles specifically have attracted great attention for implementation in clinical practice, owing to the unique physicochemical properties, such as high stability, biocompatibility and bioavailability, as well as low toxicity (Hosnedlova et al., 2018; Khurana et al., 2019; Ferro et al., 2021). It was reported that selenium nanoparticles can induce apoptosis in various cancer cell types while simultaneously safeguarding healthy cells from damage (Menon et al., 2018; Gao et al., 2020; Ferro et al., 2021). The common pathways by which selenium nanoparticles destroy cancer cells include nanoparticles internalization, regulation of reactive oxygen species production, autophagy induction and activation of intrinsic apoptotic machinery (Menon et al., 2018; Lin et al., 2021).

Given that surgical intervention is a commonly used approach for treating solid tumors, surgical site infection (SSIs) complications to breast cancer surgery are inevitable, with a reported rate as high as 19% (Williams et al., 2011; Gulluoglu et al., 2013; Olsen et al., 2015). This rate is higher than the usual expected 3.4% SSIs risk for a clean surgery category that breast cancer interventions fall into (Vazquez-Aragon et al., 2003; El-Tamer et al., 2007). The types of postoperative infections detected are monomicrobial and polymicrobial, with *Staphylococcus aureus* being the most predominant organism (Rolston et al., 2010). For this exact reason, selenium nanoparticles have gained attention owing to their potential antibacterial and anti-biofilm properties (Shakibaie et al., 2015a). It is reported that selenium nanoparticles have potential effect against problematic pathogenic bacteria, such as *Escherichia coli*, *Staphylococcus aureus* (Tran et al., 2015; Guisbiers et al., 2016) and fungi, such as *Candida albicans* (Kheradmand et al., 2014; Shakibaie et al., 2015b). This added advent of selenium nanoparticles, besides their anticancer and antioxidant activity, has raised interest in their use as a local treatment during breast cancer eradication to help control the expected postoperative infections.

Another promising anticancer element is copper (Cu). Copper plays a vital role in enzymatic activities, intracellular redox potential regulation and DNA synthesis (Daniel et al., 2005; Lopes et al., 2017; Singh et al., 2020). Its redox properties are significant in reducing toxicity, overcoming the resistance efficacy of drugs and augmenting its anticancer activity (Gunasekaran et al., 2011; Gandin et al., 2015; Singh et al., 2020). Copper accumulation, whether excessive or inadequate, influences the physiological activities of cells, with heightened demand linked to the metastasis and proliferation of tumor cells (Tsvetkov et al., 2022; Ji et al., 2023).

High initial intra-tumoral concentration and prolonged release of the active pharmaceutical ingredients (API) are made possible by *in-situ* forming implants (*ISFIs*) (Wu et al., 2017). The *ISFI* used in this study is a phase-sensitive *in-situ* forming implant. In this *ISFI* system, the drug, copper-selenium nanoparticles (Cu-Se NPs), and polymers(s) are added to an organic solvent. On injecting the *ISFI* in the body, an exchange occurs between this organic solvent and the surrounding aqueous environment, which causes precipitation of the polymer (phase inversion), resulting in a solid containing API depot that allows sustained API release for days or months. *ISFIs* provide several advantages, including the convenience of liquid administration through a catheter or needle rather than surgical interventions (Li et al., 2012; Suh et al., 2021), facilitating easy application to diverse anatomical sites (Wu et al., 2017; Young et al., 2023). *ISFIs* offer a compelling alternative for delivering high concentrations of rosuvastatin, overcoming the low bioavailability typically observed with oral administration.

To regulate the release of API from implants, poly (lactic-co-glycolic acid) (PLGA) is widely utilized as a polymeric matrix former (Shah et al., 1993; Schliecker et al., 2004; Kamel et al., 2016; Shamma et al., 2017; Kraus et al., 2018). Over the years, numerous injectable and implanted medicinal products made from PLGA have been developed. The success of this polymer can be attributed to its excellent biocompatibility, complete biodegradability into lactic acid and glycolic acid, and capacity to deliver desirable drug release rates over varying time frames, ranging from hours to several months (Elkasabgy et al., 2019; Rencber et al., 2020; Ucar et al., 2021). The market product, Zoladex^®^, is injectable PLGA vehicles used in the treatment of breast and prostate cancer by forming a matrix on injection that releases goserelin acetate (Cellesi et al., 2011).

To modify the release of API from PLGA-based *ISFIs*, an additional polymer can be combined. One of the high-performance polymers is polydimethylsiloxane (PDMS), which has special chemical and physical properties such as flexibility, thermotolerance, resistance to oxidation, ease of production and adjustable hardness, among other desirable characteristics (Jothi et al., 2019). Owing to their biocompatibility (Peterson et al., 2005), safety towards variable organisms, and biodegradability (Ceseracciu et al., 2015), PDMS are widely employed in biomedical equipment and medical implants, raising a favored soft substrate for growing various types of cells (Lee et al., 2004). Furthermore, the shell and gel of breast implants are both made of PDMS (Ramiao et al., 2017).

In this study, we leveraged the benefits of combining PLGA-based *ISFIs* with PDMS to enable localized delivery of rosuvastatin for cancer treatment in a noninvasive manner to increase the low bioavailability of this drug and achieve therapeutic medication concentrations in solid tumors. The prepared *ISFIs* were characterizes for their rheological properties, rate of matrix formation and drug release studies. Furthermore, different particle sizes of Cu-Se NPs were prepared and integrated with selected *ISFIs*. The selected *ISFIs* loaded with and without Cu-Se NPs were evaluated for their antimicrobial activity and were tested for their antitumor activity against MCF-7 and MDA-MB-231 cell lines. The combined dual effect of the proposed formulation could be advantageous for suppression of breast cancer with reduced risk of possible associated site microbial infection.

## 2 Materials and methods

### 2.1 Materials

Rosuvastatin calcium was gifted from Hikma Pharmaceuticals, Egypt. Three types of poly (D, L-lactic-co-glycolic acid) (PLGA) were kindly donated by Corbion, Amsterdam, Netherlands, with an inherent viscosity of 0.2 dL/g: (): PLGA with a 50:50 lactide to glycolide ratio (PLGA 50/50) and an ester terminal group (Purasorb^®^ PDLG 5002); (); PLGA with a 75:25 lactide to glycolide ratio (PLGA 75/25) and an ester terminal group (Purasorb^®^ PDLG 7502); and () PLGA with a 75:25 lactide to glycolide ratio (PLGA 75/25) and an acid terminal group (Purasorb^®^ PDLG 7502A).

Polydimethylsiloxanes (PDMS); Domarol DM 10000 was obtained from CISME, Milano, Italy. Dimethyl sulfoxide (DMSO) was provided by Fisher Scientific, New Hampshire, USA. Cupric nitrate (Cu (NO_3_)_2_ .3H_2_O) with a molecular weight of 241.60 g/mol was purchased from Qualikems, Nandesari, Vadodara, India. Selenium dioxide (SeO_2_) with a molecular weight of 110.96 g/mol was purchased from Sigma Aldrich, St. Louis, Missouri, USA.

Bacterial strains and culture media: strains utilized in this study included: Methicillin-resistant *Staphylococcus aureus* USA300 (MRSA USA300), which is a multiple antibiotic resistant and community-acquired strain (Diep et al., 2006), *Klebsiella pneumoniae* ATCC 13883, *Escherichia coli* K-12, and *Salmonella enterica* serovar Typhimurium ATCC 35664. The culture media used for routine culture were brain heart agar, and Mueller-Hinton agar. The control antibiotics used were gentamicin, prepared as a 40 mg/mL stock, and vancomycin, prepared as a 100 mg/mL stock.

### 2.2 Methods

#### 2.2.1 Preparation of PLGA and PDMS-based *ISFIs* loaded with rosuvastatin

As described in Table 1, the formulations for the ISFIs were prepared by dissolving the drug in 50% of the DMSO. After that, PDMS (10, 20 or 30% w/v) was added to the DMSO solution, and it was sonicated for 10 min until the formation of a homogeneous solution. Then, different types of PLGA were added at a concentration of 30% w/v, followed by DMSO to complete the final preparation volume containing 1% w/v drug. Finally, the prepared solution was sonicated for 1 hour.

**TABLE 1:** Compositions of PLGA and PDMS-based *ISFIs* loaded with rosuvastatin.

Formulation code	Compositions^a^	
	PLGA type (30% w/v)	PDMS (% w/v)
P50E1	PLGA 50/50 ester terminal	0
P50E2		10
P50E3		20
P50E4		30
P75E1	PLGA 75/25 ester terminal	0
P75E2		10
P75E3		20
P75E4		30
P75A1	PLGA 75/25 acid terminal	0
P75A2		10
P75A3		20
P75A4		30

^a^ The final concentration for rosuvastatin in the formulation was 1% w/v.

DMSO was included in each formulation to make up the remaining percentage needed to reach a total of 100% for the formulation components.

#### 2.2.2 Characterization of PLGA and PDMS-based ISFIs loaded with rosuvastatin

##### 2.2.2.1 Rheological studies

The rheological behaviors of the prepared ISFIs were investigated using a Brookfield cone and plate viscometer (DV3T rheometer, USA). The test was performed using a spindle type of CS-40 and a temperature of 25°C. Samples underwent continuous shear rate increases from 20 to 500 s^-1^ using speed intervals between 10 and 250 rpm and a time interval of 1 min between speeds. According to Farrow’s equation, the rheological behaviors of the tested *ISFIs* were measured by graphing the results of shear stress *versus* shear rate as follows (Farrow et al., 1928):

Log⁡D=NLogS−Log⁡η

where, D, N, S and *η* indicate shear rate (s^-1^), Farrow’s constant, shear stress (dyne/cm^2^) and viscosity (cP), respectively.

When the values of N equal 1, it refers to Newtonian flow, while N greater than one refers to plastic or pseudoplastic flow, and N less than one refers to dilatant flow.

##### 2.2.2.2 *In-situ* matrix formation

**FIGURE 1:**
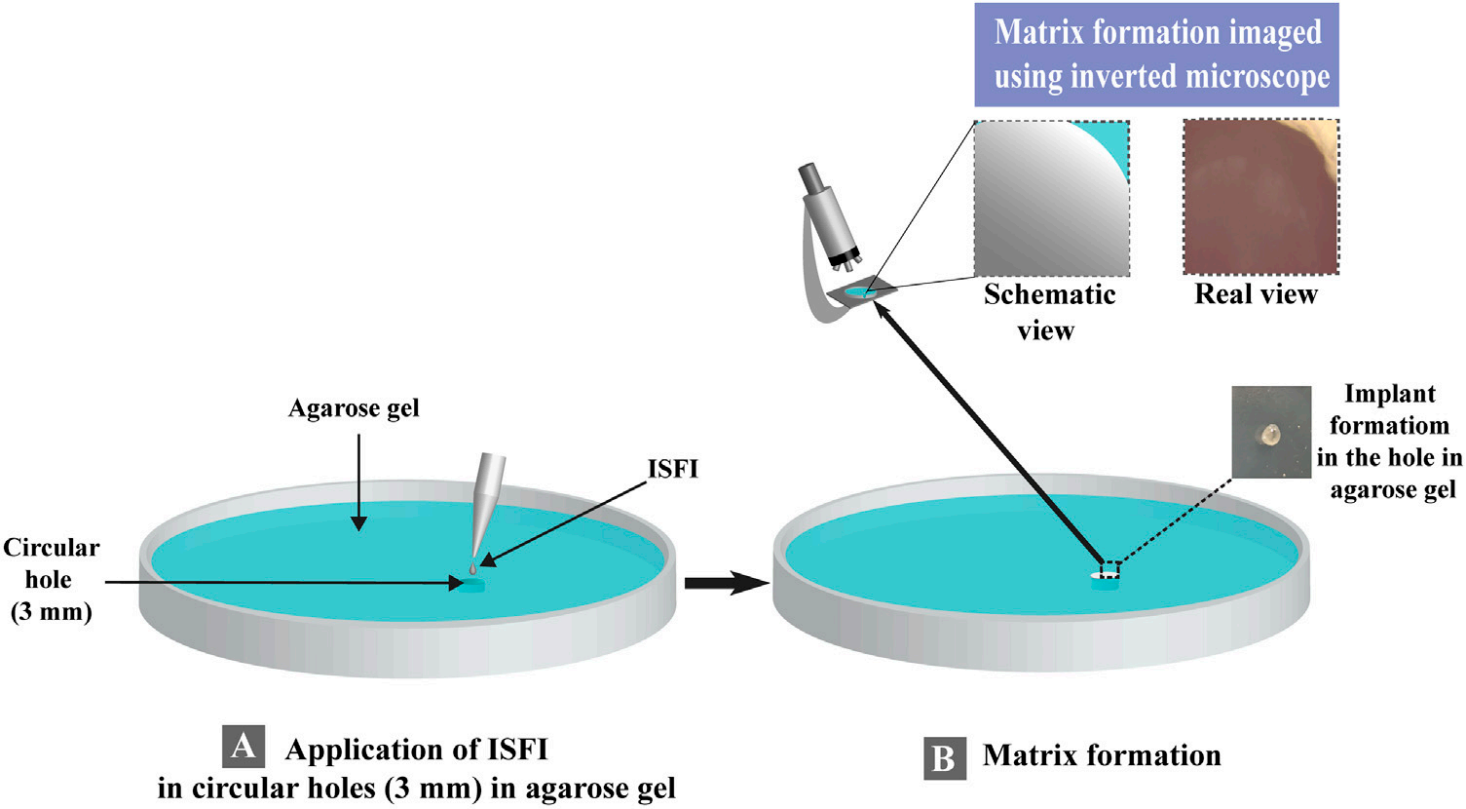
Schematic representation for the evaluation of matrix formation for *in-situ* forming implant. **(A)** ISFI Application in agarose gel and **(B)** matrix formation for *in-situ* forming implant.

Agarose gel was utilized to study the diffusion rate of water and matrix formation that mimicked the surrounding tissues after injection of the ISFI (Phaechamud et al., 2018). One hundred microliters of the prepared ISFI were added to circular holes (3 mm) in agarose gel. The appearance of an opaque matrix measuring the matrix formation of the applied *ISFI*, and if the formation of the matrix took longer than 10 min, no matrix formation data was recorded. The matrix formation was imaged using an Olympus inverted microscope (CKX41 microscope, Japan) (Figure 1). The matrix formation rate (rate of water diffusion) was estimated by applying the following equation (Phaechamud et al., 2018; Lertsuphotvanit et al., 2022):

$$MFR=\frac{D\;}{T\;}$$

where, MFR is the matrix formation rate (mm/min), D is the distance of the opaque area in the matrix (mm), and T indicates the time (min) at which the D is measured. The distance of the opaque area in the matrix was measured using specific software for image analysis named ImageJ (1.53t, NIH, USA).

##### 2.2.2.3 *In vitro* drug release

As described by Gong and his team (Gong et al., 2009), a 100 µL of the ISFI (equivalent to 1 mg of rosuvastatin) was added to Eppendorf containing 1 mL of phosphate buffer saline with a pH of 7.4 and shaken at 100 rpm (IKA Incubator shaker, KS 4000, Germany) and a temperature of 37°C. During the 3 months of drug release, 1 mL of release medium was replaced with fresh medium at each time interval. Samples were checked for rosuvastatin concentration by applying a UV-Vis spectrophotometer (Shimadzu, UV-1800, Japan) at 240 nm. KinetDS (V3.0, Poland) was used as a specific software to estimate the release efficiency (RE) values (Khan, 1975). The RE values were calculated using the following equation:

$$RE\;\left(\%\right)=\left[\left(\;\int_{0}^{t}y\times dt\right)/\left(y_{100}\times t\right)\right]\times 100$$

where, ( y ) represents the quantity of the drug released at time ( t ), while $\left(y_{100}\right)$ indicates complete drug release (100%)

#### 2.2.3 Preparation of copper-selenium nanoparticles

The utilized method herein follows a common method for the preparation of selenium–based nanoparticles (Cu-Se NPs) (Ahamed et al., 2016; Ahmed et al., 2019): 1 g of Cu(NO_3_)_2_ .3H_2_O was dissolved in 100 mL of distilled water. Subsequently, 3 g (1Cu:3Se) or 5 g of SeO_2_ (1Cu:5Se) was added to the solution, stirring until complete dissolution. The resulting solution was dried overnight at 70°C. The dried powders were subsequently characterized using X-ray diffraction (XRD), Fourier-transform infrared spectroscopy (FTIR) and ZetaSizer.

#### 2.2.4 Physicochemical characterization for the copper-selenium nanoparticles

##### 2.2.4.1 X-ray diffraction (XRD) analysis

XRD analysis (Bruker, Karlsruhe, Germany) was used to examine the crystalline nature of the produced Cu-Se NPs. The experiment was kept at a temperature of 19°C. All the specimens were scanned at a rate of 2°/min for a diffraction angle range of 2°–70°, 2θ, to take measurements. The Cu-Se NPs’ crystallinity was evaluated using the XRD-obtained curves.

##### 2.2.4.2 Fourier-transform infrared spectroscopy (FTIR) analysis

FTIR spectroscopy measurements (Demonstrate 1,600, Perkin-Elmer, Wales, UK) were taken at room temperature in the 400–4,000 cm^-1^ wavenumber range to determine the functional groups. The KBr was mixed with the nanoparticles in a ratio of 1:100 (Specimen: KBr) before being pressed into a disc in an evacuated mold.

##### 2.2.4.3 Particle size and zeta potential measurements

The particle diameter and surface charges of the Cu-Se NPs were determined by suspending 10 mg of samples in 10 mL of distilled water and analyzing them using Malvern ZetaSizer (Nano ZS, UK).

#### 2.2.5 Preparation of PLGA and PDMS-based *ISFIs* loaded with rosuvastatin and copper-selenium nanoparticles

A selected ISFIs was loaded with Cu-Se NPs at a concentration of 10 μg/mL. Then the suspension was mixed using a vortex. Two ISFI formulations were created: CS1, with Cu-Se-NPs of 560.3 nm particle size (large particle size), and CS2, with Cu-Se-NPs of 383 nm particle size (small particle size).

#### 2.2.6 Statistical analysis

The data underwent examination through a one-way ANOVA analysis. Statistical analysis was conducted using SPSS software (Version 17.0, USA). Significance was determined for results with a probability less than 0.05. Each value in this study represents the mean ± standard deviation (SD) (n = 3).

#### 2.2.7 Antimicrobial assay using agar-well diffusion

The antibacterial efficacies of the ISFIs were assessed against the following bacterial strains: MRSA USA300, *K. pneumoniae* ATCC 13883, *E. coli* K-12 and *S.* ser. Typhimurium ATCC 35664. The inoculum was prepared by culturing the bacteria on brain heart agar (BHA) (Oxoid, UK), and incubating the plates at 37°C for 24 h. After incubation, a bacterial suspension was prepared in a sterilized physiological solution to 0.5 McFarland standard (1 × 10^8^ CFU/mL). A sterile cotton swab was used to spread the inoculum suspension on 30 mL Mueller-Hinton agar (MHA).

The antimicrobial potency of the examined formulations was evaluated against the four selected organisms using the agar-well diffusion method (Perez, 1990). The tested formulation (P50E4) was loaded with both large (CS1) and small (CS2) particle size Cu-Se NPs. The CS1-M formulation was loaded with large particle size Cu-Se NPs (CS1) and rosuvastatin, making it a medicated formulation. On the other hand, CS1-NM was loaded solely with large particle size Cu-Se NPs (CS1), resulting in a non-medicated formula. Similarly, the CS2-M formulation contained small particle size Cu-Se NPs (CS2) and rosuvastatin, while CS2-NM consisted of only small particle size Cu-Se NPs (CS2), both in medicated and non-medicated formulas, respectively. Additionally, 1 w/v % rosuvastatin solution prepared in DMSO was tested separately (abbreviated by RS). A hundred mg of the mentioned formulas were added to each well (the agar gel was punctured with eight mm-diameter holes), and the plates were left on the bench for 1 hour for adequate diffusion.

The following kinds of positive controls were utilized according to the CLSI recommendation (CLSI and Wayne, 2022) (100 µL): gentamicin (8 μg/mL) for *K. pneumoniae, E. coli* K-12, *S*. ser. Typhimurium and vancomycin (8 μg/mL) for MRSA USA300. The plates were then covered with parafilm and incubated overnight under aerobic conditions at 37°C. Tests were performed in duplicates, and the forming zones of inhibition were evaluated in millimeters (mm). Statistical analysis was done using two-way ANOVA, which was followed by Tukey’s multiple comparisons test with a significance level at *p ≤* 0.05. In the graph, * means *p* ≤ 0.05, ** means *p* ≤ 0.01, *** means *p* ≤ 0.001, and **** means *p* ≤ 0.0001. The charts were generated using GraphPad Prism (v9).

#### 2.2.8 Suppression of *ISFIs* to breast cancer cells

##### 2.2.8.1 Cell culture

MDA-MB-231 breast cancer cells and MCF-7 breast adenocarcinoma cells were obtained from Nawah Scientific Research Center (Egypt). Cancer cells were incubated in a CO_2_ Cell 50 incubator (MMM Medcenter, Germany) at a temperature of 37°C with 5% v/v of CO_2_ and maintained in a specific media of DMEM containing penicillin (100 units/mL), streptomycin (100 mg/mL) and heat-inactivated fetal bovine serum in humidified (10%).

##### 2.2.8.2 Cytotoxicity assay

Cell viability was evaluated by sulforhodamine B (SRB) assay (Skehan et al., 1990; Allam et al., 2018). In 96-well plates, 100 μL cell suspension aliquots containing 5 × 10^3^ cells were incubated in complete media for 1 day. Another aliquot of 100 µL of media containing several concentrations of the tested formulations was used to treat the cells. Cells were preserved by changing the media with 150 µL of 10% trichloroacetic acid (TCA) and incubating at 4°C for 1 h after 3 days of tested formulations exposure. After removing the TCA solution, distilled water was utilized to rinse the cells five times. Volumes of 70 µL SRB solution in a concentration of 0.4% w/v were added and then incubated for 10 min at room temperature in a darkened area. Plates were thoroughly cleaned thrice with 1% acetic acid before being air-dried overnight. After dissolving the protein-bound SRB dye in 150 µL of TRIS (10 mM), an Omega microplate reader (FLUOstar, Germany) was utilized to detect the absorbance at 540 nm. The Ethics Research Committee of the Faculty of Pharmacy at Future University in Egypt, Cairo, Egypt, approved this study (REC-FPFUE-19/2023). The obtained data were statistically analyzed through a one-way ANOVA analysis as mentioned in section 2.2.6.

## 3 Results and discussion

### 3.1 Preparation of PLGA and PDMS-based ISFIs loaded with rosuvastatin

As the method described by Elkasabgy et al. (Elkasabgy et al., 2019), twelve ISFIs were prepared using PLGA as the matrix-forming material (Table 1; Figure 1). PLGA was used at a concentration of 30% w/v depending on our preliminary experiments, where 10% and 20% w/v PLGA in the ISFIs did not retard the release of rosuvastatin (unpublished data). Furthermore, PDMS was added to our formulation to monitor its effect on the different formulation properties.

### 3.2 Characterization of formulations for the PLGA and PDMS-based ISFIs loaded with rosuvastatin

#### 3.2.1 Rheological studies

Polymers can be entangled with one another through ionic interactions, hydrogen bonding, or hydrophobic interactions (Roberts and Martens, 2016). *ISFIs* prepared with PLGA having ester terminals (P50E1 and P75E1**) s**howed higher viscosity values (*p* < 0.05) in comparison with formulations prepared using PLGA having acid terminals (P75A1). The existence of methyl side groups increases the hydrophobicity of PLGA, makes its molecules connect more to each other in the hydrophilic DMSO solution, and subsequently increases the solution’s viscosity.

**FIGURE 2:**
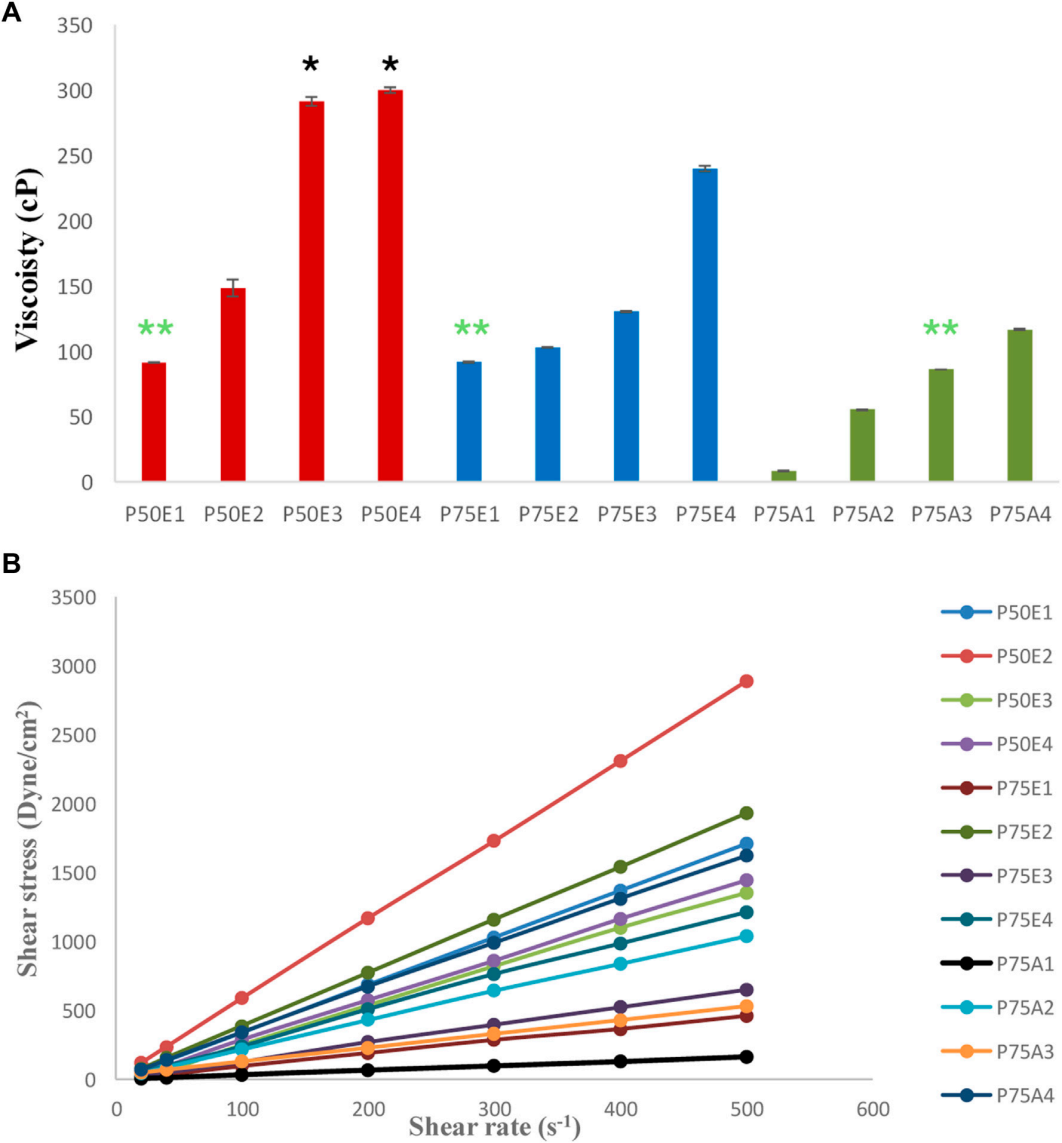
**(A)** Viscosity-values, and **(B)** rheological behaviors of the prepared *in-situ* forming implant systems. All formulations exhibited significant values (*p* < 0.05) except for the marked formulations with the same label (* and **) where non-significant values (*p* > 0.05) were observed.

Generally, it was found that increasing the concentration of PDMS in the prepared ISFIs revealed a significant increase in their viscosity values (*p* < 0.05). The increase in viscosity is related to the rise in the concentration of PDMS in the prepared system due to the decrease in the concentration of organic solvent (DMSO). Hence, the integration of PDMS in the ISFIs at a concentration of 30% w/v (the highest used concentration) demonstrated formulations with the highest viscosity values (Figure 2A).

The addition of PDMS in different concentrations (10, 20, or 30% w/v) with PLGA 50/50 with ester terminal formulations (P50E2, P50E3, and P50E4) resulted into formulations with the highest viscosity values compared to their counterparts with the same concentrations in the other formulations. This can be attributed to a specific bonding between the glycolic-rich PLGA copolymers and PDMS polymer, resulting in high-viscosity solutions.

According to Farrow’s equation, the flow pattern for all the formulations demonstrated Farrow’s constant values ranging between 0.9 and 1, referring to the Newtonian flow as shown in Figure 2B. These Farrow’s constant values reflect on easing flow and injectability of the *in-situ* gelling matrix through the syringe (Lertsuphotvanit et al., 2022).

#### 3.2.2 *In-situ* matrix formation

This study aims to completely eradicate tumor cells following the surgical treatment of cancer cells in the surrounding tissues. Therefore, a 3 mm hole in the agarose gel serves as a representation of the space created by the excised diseased tissue, where the *ISFI* was inserted. One of the essential factors in the ISFIs is the gelation time and the rate of solidification (matrix formation) upon contact with the body fluids. The rate of matrix formation depends on the water diffusion from the surrounding tissue into the ISFI, resulting in a phase inversion and solidification of the implant in the desired area. The slow rate of matrix formation may retard the solidification of the ISFIs and burst drug release, which is not favored in sustained drug release and may conflict with the aim of the *in-situ* forming systems. The matrix formation can be observed by the presence of an opaque ring and a dense matrix upon contact with the water in the agarose gel (Wang et al., 2012; Lertsuphotvanit et al., 2022).

**FIGURE 3:**
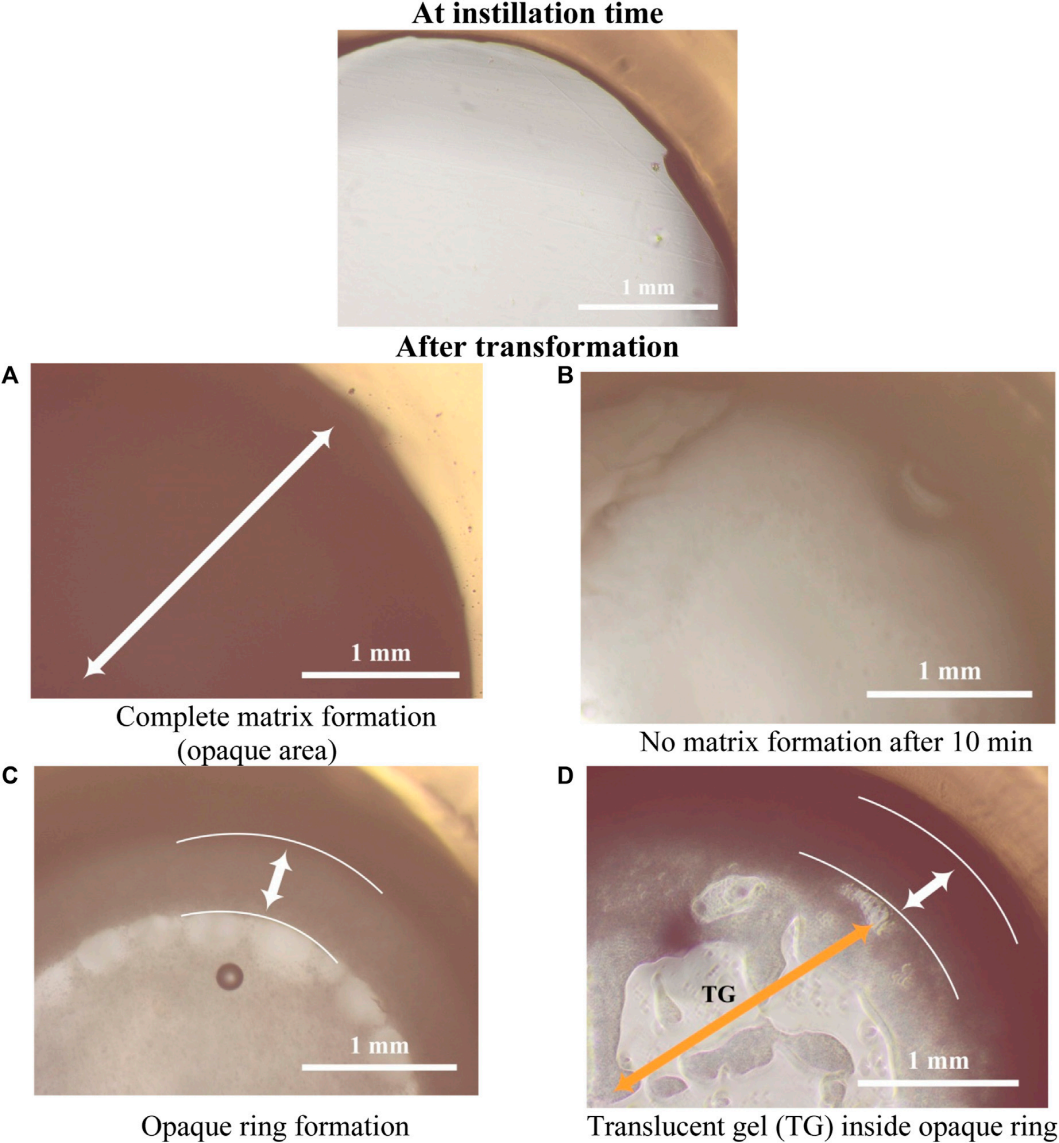
Study for *in-situ* matrix formation for *ISFIs* in agarose gel at the time of instillation into circular holes in the agarose gel and after its transformation (all images with power of magnification ×10).

**FIGURE 4:**
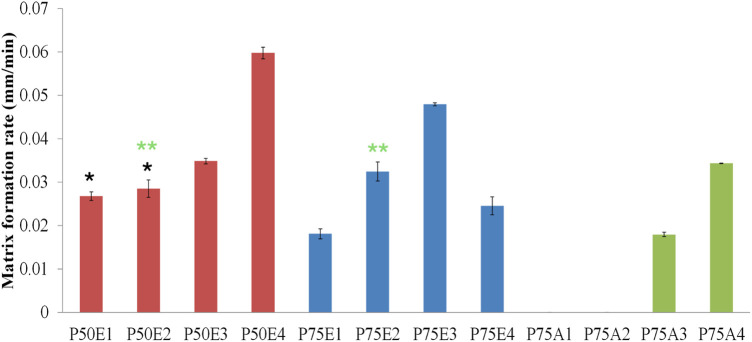
Study of the rate of matrix formation for ISFIs in agarose gel. Two statistical studies were conducted: formulations with the same PLGA concentration (designated code as P50, P75, or P75) and formulations with the same PDMS concentration (identified by 1, 2, 3, or 4 in their codes). Analyzed formulations exhibited significant values (*p* < 0.05) except for the marked formulations with the same label (* and **) where non-significant values (*p* > 0.05) were observed.

Figures 3, 4 demonstrate how the type of PLGA and concentration of PDMS had an essential effect on the rate of formulation solidification (matrix formation). In general, the rate of solidification was directly proportional to the PDMS concentration in the *ISFIs* . This mainly depended on the polymers-to-solvent ratio, where raising the polymers’ ratio resulted in rapid matrix formation upon contact with the agarose’s water and fast diffusion of the tiny amount of DMSO to the agarose, which has a high affinity to water (Elkasabgy et al., 2019).

Generally, the ISFIs prepared with PLGA 50/50 with ester terminal (P50E) revealed the highest rate of matrix formation during its contact with water in the agarose gel, followed by formulations containing PLGA 75/25 with ester terminal (P75E) and finally, formulations with PLGA 75/25 with the acid terminal (P75A) (*p* < 0.05). The rapid matrix formation achieved by PLGA 50/50-based formulation is attributed to the presence of a high concentration of glycolic acid, which has an affinity to water more than lactic acid, consequentially increasing water penetration into the formulation and rapid matrix formation (Keles et al., 2015) as shown in Figure 3A.

In the case of formulations prepared by PLGA 75/25 with acid terminal without (P75A1) and with 10% w/v PDMS (P75A2), the formulations converted into gel matrix (zero-value for rate of matrix formation; no solidification) when they were in contact with the water of agarose gel (during the 10-min study) (Figure 3B). On the other hand, the formation of an opaque ring was observed in the formulations containing 20% w/v (P75A3) and 30% w/v (P75A4) PDMS (incomplete matrix formation) as shown in Figure 3C.

Among the ISFIs containing PLGA 75/25 with an ester terminal, the opaque ring rapidly formed in all the formulations immediately upon contact with the water of agarose gel (incomplete matrix formation). Besides, increasing the concentration of PDMS from 0% w/v (P75E1) to 10% w/v (P75E2) and 20% w/v (P75E3) showed a significant increase in the rate of matrix formation during the first 10 min (*p* < 0.05), where an opaque ring is formed with a slight dense core matrix due to the diffusion of a small amount of water into the *in-situ* implant. On the other hand, a further increase in PDMS concentration to 30% w/v (P75E4) resulted in a significant decrease in the rate of matrix formation (*p* < 0.05) due to the formation of a more rigid hydrophobic ring that avoided the diffusion of water inside the implant that appears as a translucent gel core (Figure 3D), resulting in a decrease rate of matrix formation.

In summary, the formulations containing PLGA 50/50 with an ester terminal succeeded in a complete matrix formation upon contact with the water of agarose gel, especially with the addition of various concentrations of PDMS. The ISFI containing 30% w/v PDMS (P50E4) revealed the highest rate of matrix formation during the first 10 min, followed by 20% w/v PDMS (P50E3), and finally 10% w/v (P50E2) and 0% w/v (P50E1) PDMS.

#### 3.2.3 *In vitro* drug release

**FIGURE 5:**
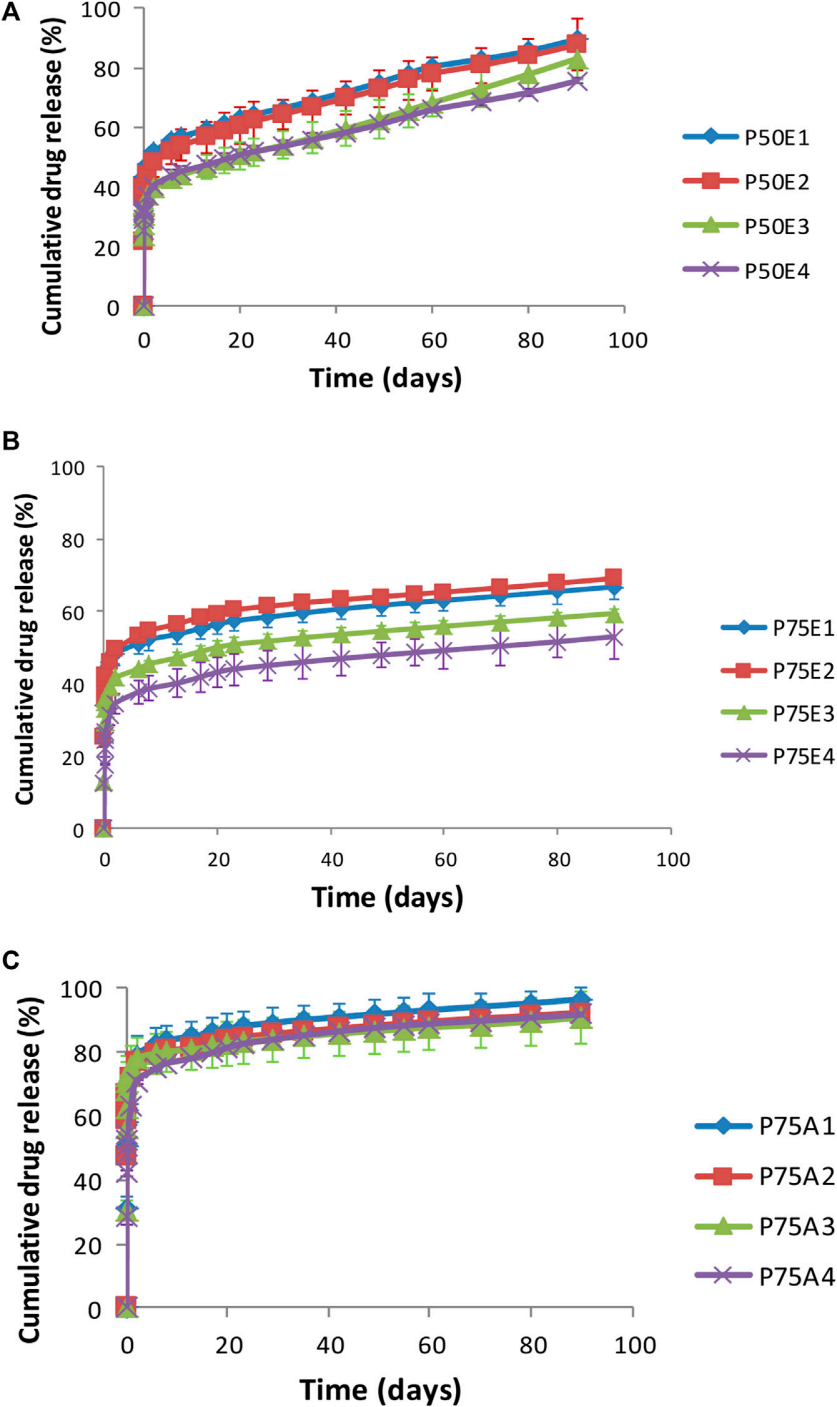
*In-vitro* drug release profiles for ISFIs containing **(A)** PLGA 50/50 with ester terminal (P50E), **(B)** PLGA 75/25 with ester terminal (P75E) and **(C)** PLGA 75/25 with an acid terminal (P75A).

Table 2 and Figure 5 illustrate the *in-vitro* drug release data and profiles from the ISFIs. In most cases, the formulations demonstrated a gradual drug release over the 3-month study period. Generally, the formulations containing PLGA 75/25 with ester terminal (P75E) revealed the lowest release efficiency-values and slowest drug release, followed by formulations containing PLGA 50/50 with ester terminal (P50E), and finally, the fastest drug release was from formulation containing PLGA 75/25 with acid terminal (P75A) (*p* < 0.05). This can be attributed to the fact that PLGA with an ester terminal (P75E and P50E) is more hydrophobic than PLGA with an acid terminal (P75A) which retards drug release (Youshia et al., 2017; Rafiei and Haddadi, 2019). Furthermore, the ratios of lactic acid to glycolic acid in an intrinsic factor to determine the hydrophobicity of PLGA polymers (Su et al., 2021), where increasing lactic acid increases the hydrophobicity of PLGA 75/25 (P75E) compared to PLGA 50/50 (P50E) with a lower concentration of lactic acid, resulting in a more hydrophobic *ISFI* and retard drug release upon direct contact with the release media.

**TABLE 2:** Release efficiency values for *ISFIs* loaded with rosuvastatin.

Formulation code	Release efficiency^a^ (%)
P50E1	71.9 ± 0.6
P50E2	69.6 ± 5.8
P50E3	61.7 ± 3.8
P50E4	58.6 ± 1.6
P75E1	59.5 ± 2.8
P75E2	62.0 ± 0.0
P75E3	52.6 ± 1.5
P75E4	46.3 ± 3.5
P75A1	89.7 ± 4.6
P75A2	86.2 ± 0.5
P75A3	84.9 ± 6.9
P75A4	84.5 ± 0.5

^a^ Each value represents the mean ± SD (n = 3).

On the other hand, the *ISFI* formulations containing PLGA 75/25 with an acid terminal (P75A) prepared with and without PDMS showed no significant difference in their release efficiency values (*p* > 0.05), where their values exceeded 80%. This may be due to the possibility that carboxyl terminal groups can stimulate the hydrolysis of ester bonds, producing additional acidic groups and generating an autocatalytic cycle that speeds up polymer degradation (Hua et al., 2021).

It was observed that raising the concentrations of PDMS in the formulations prepared using PLGA 75/25 with ester terminal (P75E) revealed a significant retardation in drug release (*p* < 0.05). The increase in the concentration of PDMS incorporated with PLGA 75/25 having an ester terminal resulted in a more hydrophobic ISFI, resulting in the formation of a dense matrix upon contact with the release medium, where the drug is entrapped inside the dense matrix, and the release of the drug depends on the degradation of the formed implant. This explains why the percentage of drug release did not exceed 60% after 3 months of the drug release study. The ISFI containing 30% w/v PDMS (P75E4) demonstrated the lowest release efficiency value, followed by the formulation containing 20% w/v PDMS (P75E3), and finally, the formulation containing 10% w/v (P75E2) and formulation prepared without PDMS (P75E1) (*p* < 0.05). This may be correlated to the increase in matrix formation rate caused by increasing PDMS concentration to 10% and 20% w/v in P75E. Although P75E4 gave a smaller value for matrix formation rate compared to P75E1, P75E2 and P75E4, but the solidified shell was able to retard the drug release.

Formulations containing PLGA 50/50 with ester terminals (P50E) showed a gradual drug release pattern over the studied period (3 months) in comparison to other tested formulations. This can be referred to as the fast matrix formation rate for such formulations, through which the drug should diffuse gradually to reach the release medium. Furthermore, the addition of PDMS to the formulations in a concentration of 20% w/v (P50E3) and 30% w/v (P50E4) showed a significant decrease in their release efficiency-values compared to the formulations prepared without the PDMS (P50E1) and formulation prepared with 10% w/v PDMS (P50E2) (*p* < 0.05). The retardation of drug release in high concentrations of PDMS can be due to the increase in the hydrophobicity of the *in-situ* gelling system and the high implant formation rate upon contact with water, avoiding the initial burst effect of the drug. These results confirmed that the rapid matrix formation could decrease the initial burst effect and sustain drug release.

All the *ISFIs* exhibited the best fit to the Korsmeyer-Peppas model of drug release, with regression coefficient values (r^2^) exceeding 0.91. Additionally, the *ISFIs* showed diffusional exponent (n) values of less than 0.45, indicating that the release mechanism of rosuvastatin from the formulations follows Fickian diffusion release.

From the previous studies, we found that P50E formulations had the ability to release the drug in a gradual manner, and P50E3 and P50E4 had the lowest release efficiency-values. Furthermore, both formulations had the same viscosity-values, but P50E4 had a larger value for matrix formation rate, which ensures fast implant formation after injecting the formulation and thus prevents its dilution from the surrounding body fluids. Therefore, the P50E4 formulation was nominated as the best *in-situ* gelling implant.

### 3.3 Physicochemical characterization for the copper-selenium nanoparticles

#### 3.3.1 XRD analysis

**FIGURE 6:**
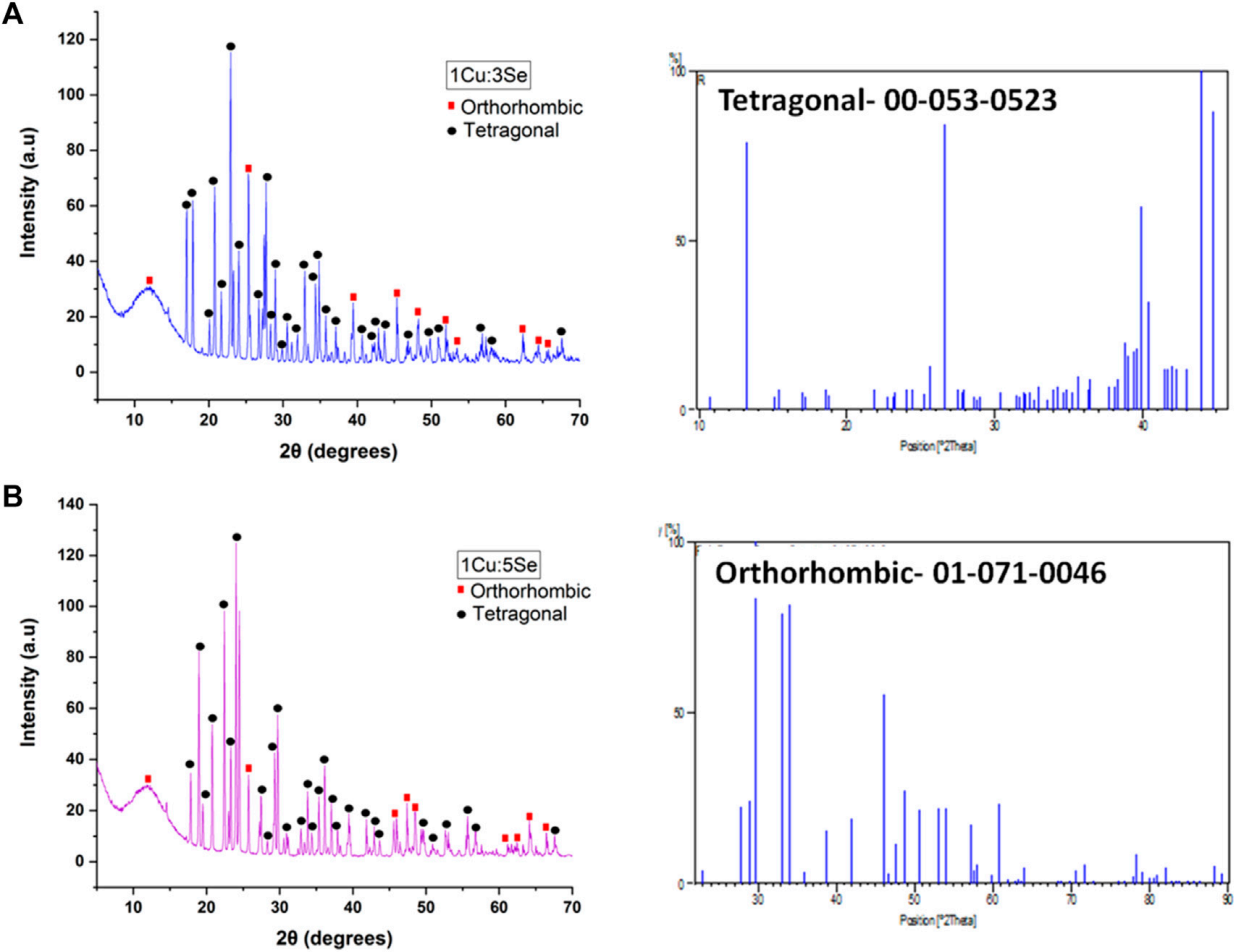
XRD of the prepared copper-selenide nanoparticles **(A)** 1Cu:3Se (CS1) and **(B)** 1Cu:5Se (CS2); with their most matching cards.

Figure 6 shows the XRD patterns of the fabricated nanoparticles. The presence of copper and selenium was confirmed in the fabricated nanoparticles by observing the XRD pattern of the Cu-Se NPs (Orthorhombic), which showed distinctive features corresponding to peaks 11.5, 21.0, 25.5, 30.0, 39.3, 45.5, 47.0, 51.5, 52.0, 62.3, 64.5 2θ with the following planes (030), (200), (250), (211), (006), (Huang et al., 2016), (620), (511), (541), (Warita et al., 2014; Anzar et al., 2018), (701), (920), and (940), which is in excellent agreement with the Cu_2_Se_x_ (P) structure as determined by the Joint Committee on Powder Diffraction Standers (JCPDS), cards numbers (00-47-1,448, 01-071-0046 and 01-074-0280) of Orthorhombic Cu_2_Se_x_ (Kumar and Singh, 2011)*.* However, some other peaks were observed in the XRD patterns of nanoparticles, which might be explained by the presence of tetragonal Cu_3_Se_2_ (20.5, 23.0, 27.0, 28.5, 29.0, 32.5, 37.0, 41.5, 43.0, 45.0, 47.0, 50.0, 51.0, 53.0, and 55.0 2θ) that matches cards No.03- 065-1,656 and 00-053-0523 (Qiao et al., 2017). From both XRD curves of 1Cu:3Se (CS1) and 1Cu:5Se (CS2), it is obvious that tetragonal Cu-Se NPs is the dominant phase for sample 1Cu:3Se while the increment in Se concentration (1Cu:5Se) has increased the orthorhombic phase on the expense of the tetragonal one. It is worth highlighting that nanomaterials’ physicochemical, mechanical properties and anticancer activity can be significantly influenced by their geometric characteristics, such as orthorhombic or tetragonal arrangements. Their geometry influences nanomaterials’ surface area, surface charge, and crystal structure, impacting their physicochemical properties. The effects of orthorhombic and tetragonal nanomaterials on cancer cells have been the subject of numerous investigations. One instance of investigation involves the utilization of orthorhombic nanoparticles that include customized surface chemistry for the purpose of targeted drug delivery, as they have demonstrated enhanced cellular uptake and heightened cytotoxic effects against cancer cells. Conversely, tetragonal nanomaterials have been examined for their capacity to function as photothermal agents. Their distinctive structure enables effective absorption of light and conversion into heat, resulting in the targeted removal of tumors (Yin et al., 2013; Chen et al., 2022).

#### 3.3.2 FTIR analysis

**FIGURE 7:**
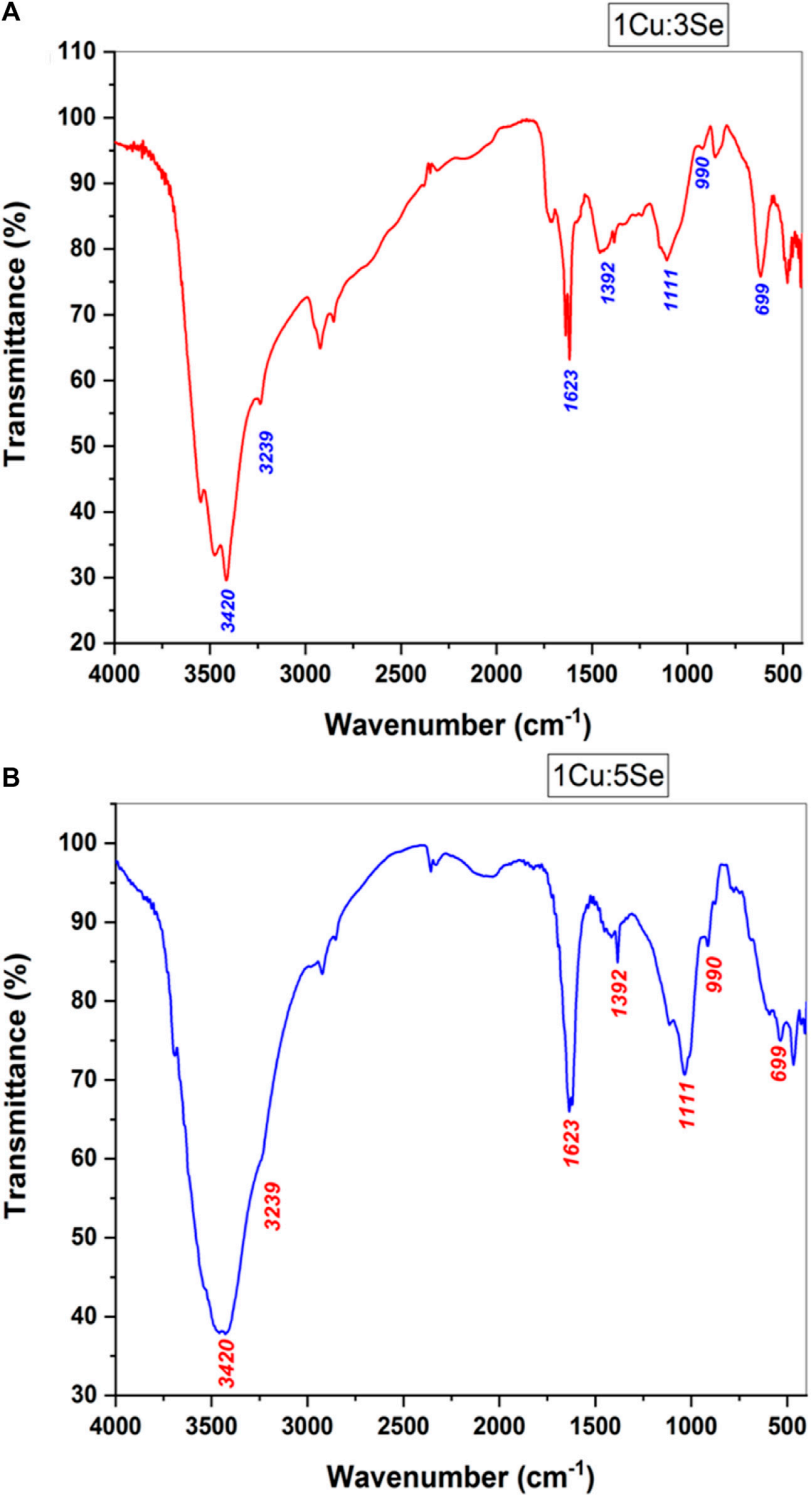
FTIR of **(A)** 1Cu:3Se (CS1) and **(B)** 1Cu:5Se (CS2) nanoparticles.

Figure 7 represents the FTIR curves of the prepared Cu-Se NPs. The FTIR spectra of the fabricated nanoparticles possess the main characteristic bands of copper-selenide (Nouri et al., 2017). In detail, bands located at 3,420 cm^-1^ and 3,239 cm^-1^ correspond to the -OH stretching vibration functional group, which might be due to the adsorbed water on the samples from the surrounding atmosphere (Ahmed et al., 2019; Mabrouk et al., 2022). Generally, bands in the 2,800-3,000 cm^-1^ range represent alkene (C–H) stretch vibrations. The presence of the O-H stretch vibration group was presented at 1,623 cm^-1^, and the band related to aromatic (C=C) bonds was observed with a medium group that was noted at 1,392 cm^-1^. These bands are suggested to be related to some adsorbed element from the surrounding environment as well, as it was highlighted earlier in our previous researches (Ahmed et al., 2019; Mabrouk et al., 2021), especially that Cu-Se NPs were prepared at room temperature. The adsorption of water and/or gases onto the surface of nanomaterials is a result of their large surface area and their geometries. The gas adsorption capabilities of nanomaterials can be significantly improved when they possess well-defined geometries, such as orthorhombic or tetragonal structures, in comparison to their bulk counterparts (Sadegh et al., 2017; Baig et al., 2021; Ahmed et al., 2022). Bands observed at 1,111 cm^-1^, 990 cm^-1^ and 699 cm^-1^ are normally correlated to the presence of inorganic functional groups such as Cu and Se.

#### 3.3.3 Particle size and zeta potential measurements

The particle size of the obtained Cu-Se NPs was shown to be in the nano-range. Mainly, sample 1Cu:3Se (CS1) recorded a size of 560.3 ± 9.6 nm with a polydispersity index (PDI) value of 0.532, and sample 1Cu:5Se (CS2) possessed a particle diameter of 383.6 ± 18.3 nm with a PDI value of 0.387. This suggested that the presence of Se in high concentration (1Cu:5Se) enhances the repulsive force between the particles and decreases their agglomeration, resulting in a smaller final particle diameter than 1Cu:3Se. Furthermore, the zeta potential values were less than ±5 and were considered as neutral (−1.92 mV for 1Cu:3Se NPs and 0.355 mV for 1Cu:5Se NPs). The observations strongly suggest a complete interaction between selenium and copper ions in the given ratios. Given the low zeta potential, it is advisable to conduct a stability assessment to determine optimal storage conditions and an appropiate container for the final formulation, such as an easily mixable syringe system.

1Cu:3Se nanoparticles with larger particle sizes (CS1) and 1Cu:5Se nanoparticles with smaller particle sizes (CS2) were suspended (10 μg/mL) into the selected formulation P50E4 to prepare P50E4 (CS1) and P50E4(CS2) formulations, respectively.

### 3.4 Antimicrobial assay using agar-well diffusion

Surgical site infections are one of the hurdles accompanying breast cancer surgery, with rates higher than those normally reported for clean surgeries. This necessitates controlling using materials with good coverage for the reported resistant organisms. One of the emerging promising nanomaterials in the health field and biomedical devices are nanoparticles, and metal associated ones specifically, owing to their promising inhibitory action against problematic pathogenic microorganisms, specifically those reported in postoperative infections of breast cancer surgery cases (Huang et al., 2016).

**FIGURE 8:**
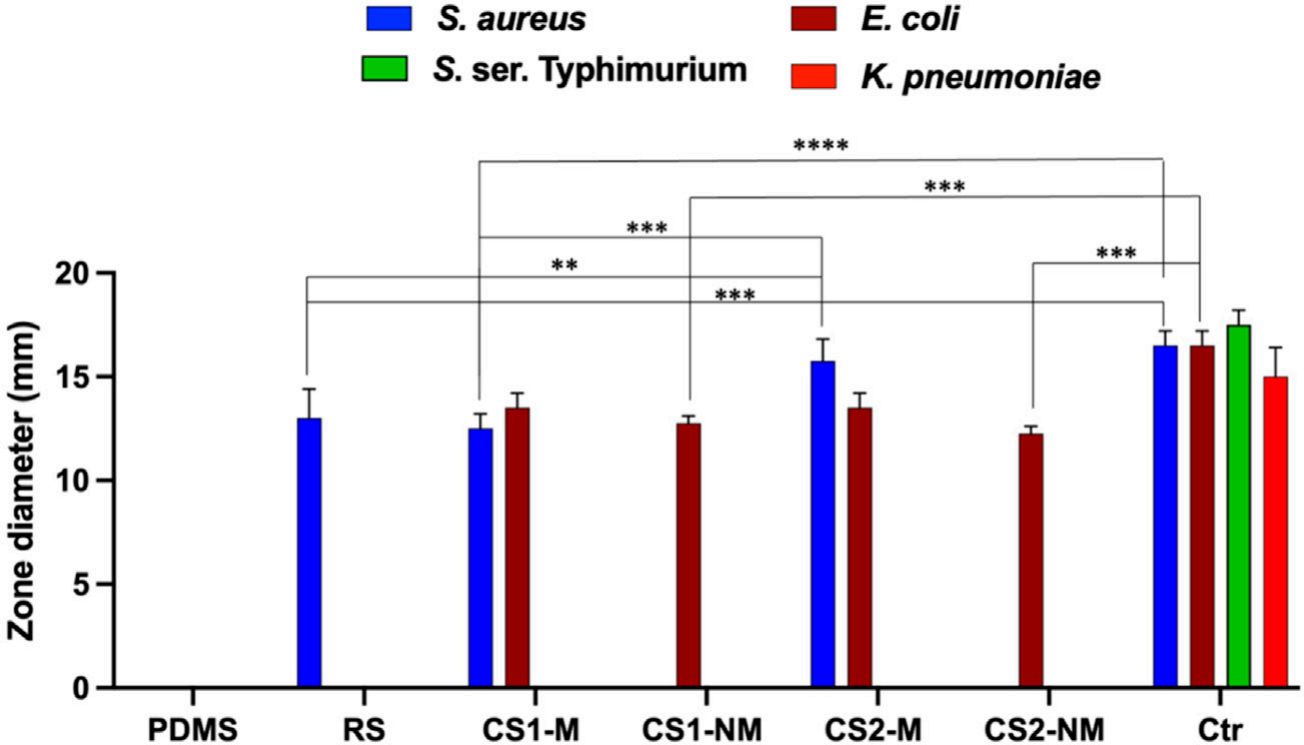
A bar chart showing the antimicrobial activity of the four tested formulas in terms of inhibition zone diameter. The strains used were *Staphylococcus aureus* USA300, *Klebsiella pneumoniae* ATCC 13883, *Escherichia coli* K-12 and *S.* ser. Typhimurium ATCC 35664. Gentamycin was used as a control for Gram negative organisms, while vancomycin was used as the control for Gram positive ones. Abbreviations: PDMS; polydimethylsiloxane, RS; 1 w/v % rosuvastatin solution prepared in DMSO, CS1-M; 10 μg/mL large particle size Cu-Se NPs and 1 w/v % rosuvastatin in P50E4, CS1-NM; 10 μg/mL large particle size Cu-Se NPs only in P50E4, CS2-M; 10 μg/mL small particle size Cu-Se NPs and 1 w/v % rosuvastatin in P50E4, CS2-NM; 10 μg/mL small particle size Cu-Se NPs only in P50E4, Ctr; control.

First, PDMS was tested in a concentration similar to that incorporated in formula (30% w/v), and it showed no inhibitory effect on the tested strains (Figure 8. PDMS). PLGA was also tested and likewise, it showed no inhibitory effect on the tested strains (Not shown on the graph). When the rosuvastatin was tested alone, it showed an inhibitory effect on MRSA USA300, a highly resilient strain that has been reported globally, both in community and healthcare settings (Enström et al., 2018) (Figure 8. RS); however, the vancomycin control showed statistically more significant inhibition (*p* ≤ 0.001).

Upon testing the non-medicated formulations containing Cu-Se NPs (CS1-NM and CS2-NM), they both showed antibacterial activity against *E. coli* K-12 only (among the tested organisms) and the difference between them was not statistically significant; however, the gentamycin control showed statistically more significant inhibition compared to both non-medicated formulations (*p* ≤ 0.001). This is in line with reports about the antimicrobial properties of selenium in nanoparticle form, owing to its size and charge (Geoffrion et al., 2020). The tested medicated formulations (CS1-M and CS2-M) had an antibacterial potency against both *E. coli* K-12 and MRSA USA300 due to the presence of Cu-Se NPs and rosuvastatin, respectively.

The Cu-Se NPs with smaller particle size (CS2-M) significantly increased the inhibition zone towards MRSA USA300 compared to the larger sized particles (CS1-M) (*p* ≤ 0.001) and compared to the rosuvastatin alone (*p* ≤ 0.01), which highlights the effect of the Cu-Se NPs. Additionally, CS2-M was the only formula that had no significant difference in the zone diameter compared to the vancomycin control (*p* > 0.05). Upon testing the antibacterial activity against *S.* ser. Typhimurium, a decrease in the growth density zone was observed rather than a clear bacterial inhibition with the medicated formulations loaded with both sizes of nanoparticles (CS1-M and CS2-M). This zone is regarded as a result of hetero-resistance, which is a phenomenon demonstrated by a subset of a microbial population that is often thought to be susceptible (El-Halfawy and Valvano, 2015).

### 3.5 Suppression of ISFIs to breast cancer cells

**FIGURE 9:**
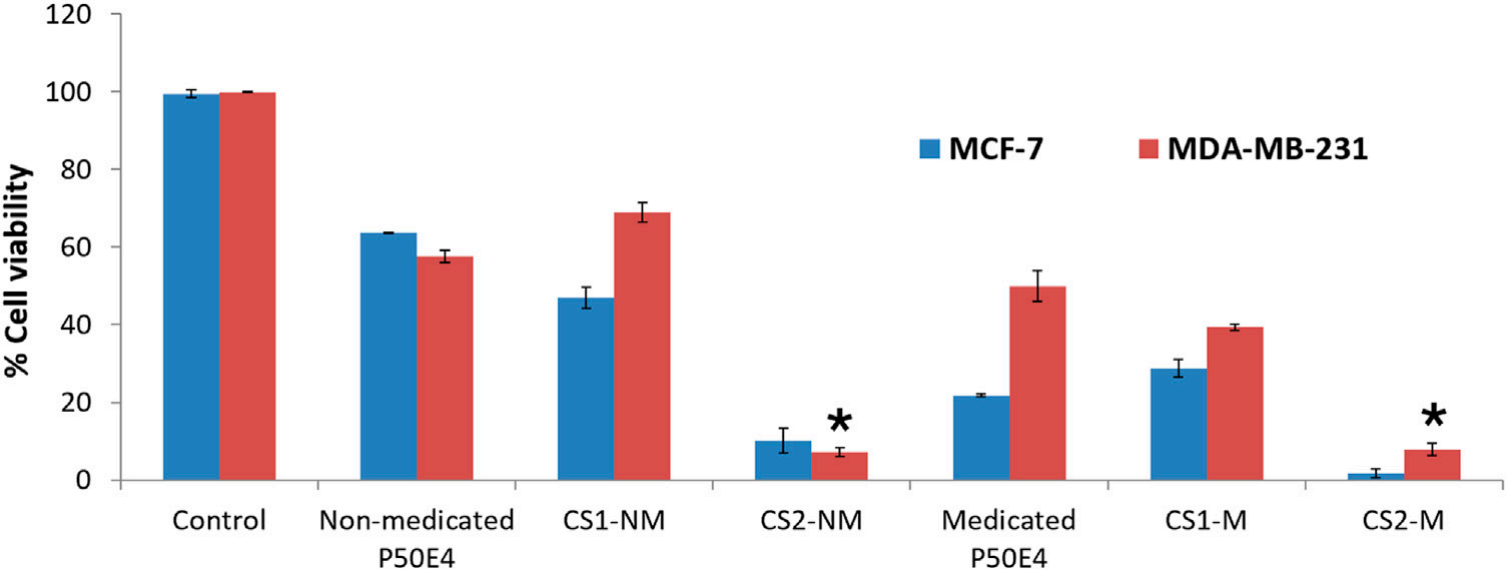
Cell viability evaluation of selected formulation P50E4 on breast cancer cell lines (MCF-7 and MDA-MB-231). Abbreviations: CS1-M; 10 μg/mL large particle size Cu-Se NPs and 1 w/v % rosuvastatin in P50E4, CS1-NM; 10 μg/mL large particle size Cu-Se NPs only in P50E4, CS2-M; 10 μg/mL small particle size Cu-Se NPs and 1 w/v % rosuvastatin in P50E4, CS2-NM; 10 μg/mL small particle size Cu-Se NPs only in P50E4, Ctr; control. All formulations exhibited significant values (*p* < 0.05) except for the marked formulations (*) where non-significant values (*p* > 0.05) were observed.

**FIGURE 10:**
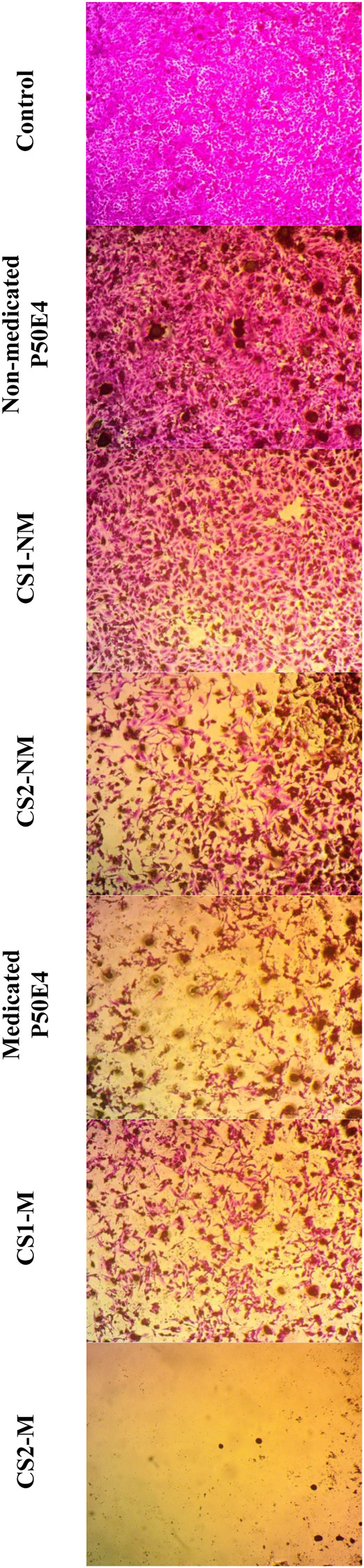
Inverted microscope images of MCF-7 cell line treated with different tested formulations. Abbreviations: CS1-M; 10 μg/mL large particle size Cu-Se NPs and 1 w/v % rosuvastatin in P50E4, CS1-NM; 10 μg/mL large particle size Cu-Se NPs only in P50E4, CS2-M; 10 μg/mL small particle size Cu-Se NPs and 1 w/v % rosuvastatin in P50E4, CS2-NM; 10 μg/mL small particle size Cu-Se NPs only in P50E4, Ctr; control.

**FIGURE 11:**
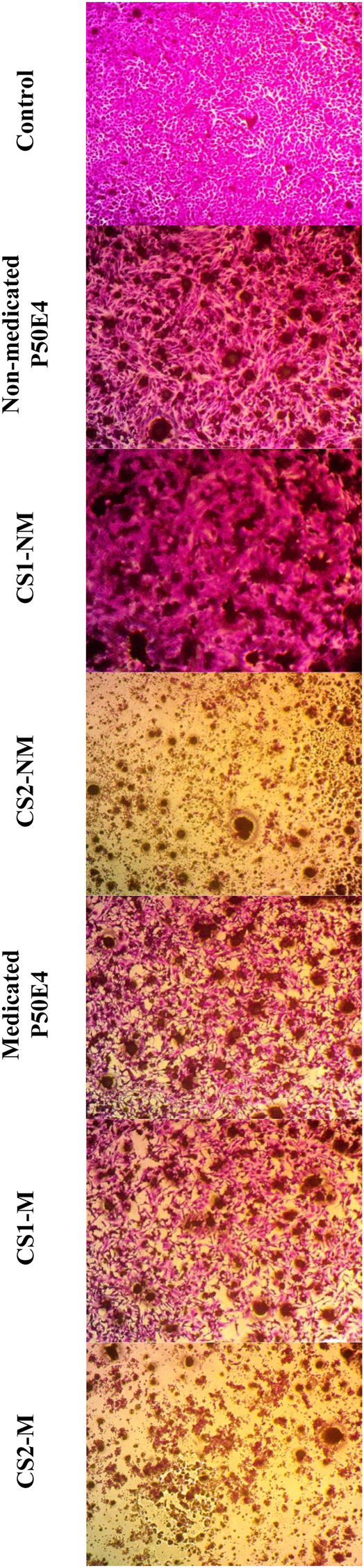
Inverted microscope images of --MDA-MB-231 cell line treated with different tested formulations. Abbreviations: CS1-M; 10 μg/mL large particle size Cu-Se NPs and 1 w/v % rosuvastatin in P50E4, CS1-NM; 10 μg/mL large particle size Cu-Se NPs only in P50E4, CS2-M; 10 μg/mL small particle size Cu-Se NPs and 1 w/v % rosuvastatin in P50E4, CS2-NM; 10 μg/mL small particle size Cu-Se NPs only in P50E4, Ctr; control.

The efficacy against breast cancer cell lines (MCF-7 and MDA-MB-231) of non-medicated and medicated P50E4 formulations loaded with or without Cu-Se NPs was evaluated using SRB assay, as shown in Figures 9–11.

In MCF-7 cell lines, primarily, non-medicated P50E4 exhibited a significant reduction in cell viability compared to the untreated control (*p* < 0.05), with cell viability decreasing to 63%. This decrease may be attributed to the unfavorable adherence, spreading, and proliferation of breast cancer cells on PDMS (Zhang et al., 2013). The addition of Cu-Se NPs to ISFIs (CS1-NM and CS2-NM) showed a significant decrease in cell viability compared to the control, which did not receive any treatment (*p* < 0.05), where the percentage of cell viability decreased from 99.4% to values less than 46.97%. Cu-Se NPs have the affinity to bind to the wall and DNA of the cancer cells through electrostatic interaction, as copper ions bind with nitrogen present in the DNA of MCF-7, resulting in DNA damage, dysfunction of mitochondria, spoiling of metabolic pathways and cell death through oxidative stress (Antony et al., 2022). On the other hand, CS2-NM and CS2-M, containing smaller particle sizes of Cu-Se NPs (CS2), demonstrated a significant suppression in cell viability of cancer cells compared to the CS1-NM and CS1-M (*p* < 0.05); this explains that the smaller particle sizes have the opportunity to pass the cell membrane of cancer cells easily than the larger particle sizes (Anzar et al., 2018). Medicated P50E4 formulation demonstrated an anticancer activity and decrease in cell viability in comparison to non-medicated P50E4 formulation (*p* < 0.05); subsequently, adding rosuvastatin to Cu-Se NPs formulations boosted anticancer efficacy by over 1.5 and 28 times when compared to non-medicated formulations with larger (CS1-M) and smaller particle sizes (CS2-M) (*p* < 0.05), respectively. Such results prove the anticancer activity of rosuvastatin and its synergistic effect, especially when incorporated with the small particle sizes of Cu-Se NPs (CS2-M) to act as tumor-suppressive. Additionally, it has been reported that rosuvastatin exhibits inhibitory effects on the HSC-3 human tongue squamous carcinoma cell line (Hosny et al., 2020).

In the MDA-MB-231 cell line, CS1-NM showed no superior inhibitory impact on cell viability compared to non-medicated P50E4 (*p* < 0.05). Conversely, the medicated formulations P50E4 and -CS1-M reduced cell viability by 1.4 and 1.8 fold, respectively, compared to non-medicated P50E4. The inclusion of small particle sizes of Cu-Se NPs in CS2-NM and CS2-M led to a significant decrease in cell viability by 9.5 and 8.6 fold, respectively, compared to non-medicated P50E4 (*p* < 0.05). Incorporating rosuvastatin into CS1-NM led to a notable decrease in cell viability (CS1-M; *p* < 0.05), whereas its addition to CS2-NM did not result in any additional effect on reducing cell viability (CS2-M; *p* > 0.05). It was observed that *ISFIs* exhibited lower sensitivity in reducing cell viability in MDA-MB-231 compared to MCF-7 (*p* < 0.05), potentially due to the partial resistance of statin invasion caused by the exogenous E-cadherin expression on the cell surface of MDA-MB-231 (Warita et al., 2014). Further assessment of the anticancer effects of the formulation and its components on additional types of breast cancer is advised, as each subtype may exhibit a distinct response to our formulation.

Regarding Cu-Se NPs toxicity to normal cells, several research studies (Pramanik et al., 2016; Jaidev et al., 2017; Rivera et al., 2021; Alasvand et al., 2023) have confirmed low or minimal release of free copper from complexes and polymer matrices (Menon et al., 2018; Gao et al., 2020; Ferro et al., 2021). Additionally, the copper-to-selenium ratios are low (1:3 or 1:5) compared to selenium, which will also contribute to a decrease in the expected release of free copper. Additionally, studies have reported that selenium nanoparticles can induce apoptosis in different types of cancer cells, while safeguarding healthy cells from harm. Puri *et al.* reported that selenium nanoparticles exhibited low toxicity to normal cells and increased antimicrobial and antitumor efficacy against cancer cell line (MCF-7). Selenium nanoparticle doses of 50–100 μg/mL caused only 4% lysis of red blood cells (representing normal cells in the study). (Puri et al., 2023).

The previous results indicate that adding Cu-Se NPs to the formulations inhibits the growth of breast cancer cell lines (MCF-7 and MDA-MB-231). The smaller particle sizes of Cu-Se NPs (CS2) showed greater cell viability suppression than the larger particle sizes (CS1). Additionally, rosuvastatin demonstrates anticancer activity and decreased cell viability. The combination of rosuvastatin with Cu-Se NPs further enhanced the anticancer activity, particularly with the smaller particle sizes (CS2-M).

## 4 Conclusion

*ISFIs* loaded with rosuvastatin were prepared using three PLGA types and different PDMS concentrations (0, 10, 20 and 30%). The prepared ISFIs were evaluated for their rheological behaviors, *in-situ* forming implant’s matrix rate and *in-vitro* study for rosuvastatin release. The rheological behavior of the prepared *in-situ* implants showed a Newtonian flow. The nominated formulation containing PLGA 50/50 with an ester terminal with 30% PDMS showed the fastest matrix formation rate with a release efficiency of 58.6%. On the other hand, Cu-Se NPs were prepared in weight ratios of 1:3 and 1:5 Cu to Se, and evaluated by XRD, FTIR and ZetaSizer. Their particle size values were 560.3 and 383.6 nm for nanoparticles with weight ratios of 1:3 and 1:5 Cu to Se, respectively. Advantageously, the tested medicated formulations showed an antibacterial potency against *Escherichia coli* K-12 and MRSA USA300. Rosuvastatin-loaded implant revealed antitumor efficacy against cancer cell lines (MCF-7 and MDA-MB-231), and the integration of small particle size of Cu-Se NPs with rosuvastatin showed a synergetic antitumor activity towards the cancer cell lines. Cu-Se NPs with smaller particle size showed superior antibacterial activities and antitumor efficacy. Further investigations utilizing animal models are warranted to comprehensively evaluate the therapeutic efficacy of the formulation. These studies would facilitate the determination of safety profiles, identification of optimal dosages, and establishment of suitable treatment regimens. Moreover, such research endeavors will provide valuable insights into potential toxicities to normal cells, thus addressing the concerns raised regarding *in vivo* application.

## Data Availability

The raw data supporting the conclusion of this article will be made available by the authors, without undue reservation.
